# Worldwide 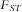 Estimates Relative to Five Continental-Scale Populations

**DOI:** 10.1111/ahg.12081

**Published:** 2014-09-17

**Authors:** Christopher D Steele, Denise Syndercombe Court, David J Balding

**Affiliations:** 1 UCL Genetics Institute, Darwin Building Gower StreetLondon, WC1E 6BT, UK; 2 Analytical & Environmental Sciences, Kings College, London, Stamford StreetLondon, SE1 9NH, UK

**Keywords:** Microsatellite, short tandem repeat, *F_ST_*, fixation index, forensic

## Abstract

We estimate the population genetics parameter 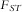 (also referred to as the fixation index) from short tandem repeat (STR) allele frequencies, comparing many worldwide human subpopulations at approximately the national level with continental-scale populations. 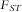 is commonly used to measure population differentiation, and is important in forensic DNA analysis to account for remote shared ancestry between a suspect and an alternative source of the DNA. We estimate 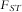 comparing subpopulations with a hypothetical ancestral population, which is the approach most widely used in population genetics, and also compare a subpopulation with a sampled reference population, which is more appropriate for forensic applications. Both estimation methods are likelihood-based, in which 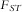 is related to the variance of the multinomial-Dirichlet distribution for allele counts. Overall, we find low 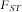 values, with posterior 97.5 percentiles 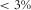 when comparing a subpopulation with the most appropriate population, and even for inter-population comparisons we find 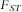 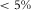. These are much smaller than single nucleotide polymorphism-based inter-continental 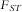 estimates, and are also about half the magnitude of STR-based estimates from population genetics surveys that focus on distinct ethnic groups rather than a general population. Our findings support the use of 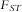 up to 3% in forensic calculations, which corresponds to some current practice.

## Introduction

We analyse an extensive new data set of the short tandem repeat (STR) profiles of individuals with worldwide origins, to estimate 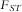 for national-scale subpopulations relative to continental-scale populations. We use two approaches to estimating 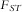, which differ according to the choice of reference population: a direct method that is appropriate for forensic applications, and an indirect method that reflects current population genetics practice.

In a forensic setting, 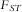 is used to account for distant relatedness (coancestry) between the queried contributor (Q) and the unknown individual X that replaces Q in the defence hypothesis (Weir, 2007). Larger values of 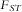 imply greater coancestry and so a greater probability that the profiles of X and Q are similar. This results in a lower likelihood ratio (LR), meaning that ignoring coancestry between X and Q is unfavourable to the defendant. The difference is unimportant for full-profile matches because even after 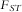 adjustment the resulting LR is extremely large, and may be rounded down for example to 1 billion for reporting in court. However, 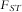 adjustments are widely used, and can have a substantial impact, in analyses of mixed and low-template DNA profiles. The use of an 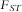 adjustment can be regarded as allowing for additional uncertainty arising from the fact that the available database does not fit the circumstances of the case perfectly, which logically reduces confidence in the result, reflected in the reduced LR.

The appropriate value of 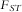 in forensic work is relative to the reference database used, and may therefore differ substantially from 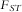 estimates arising in population genetics research. Even if Q and X have a very similar ethnic background, a low 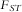 value may suffice if the allele frequency database is directly appropriate for both Q and X, whereas the more distant they are from the database population, the larger the 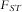 value that is required (Steele & Balding, 2014). It is usually regarded as reasonable to give the defence some benefit of doubt and to apply a generous 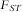 value to all possible X drawn from the same population as Q. If, on the other hand, Q is Caucasian and we wish to consider an X who is Afro-Caribbean, then the Afro-Caribbean database is appropriate and since little coancestry is expected between Q and X relative to this database, only a low value of 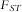 would be required. There is always some uncertainty about the appropriate 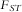 values: there is the usual variation in any statistical estimate but we have additional uncertainty here because 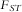 is rarely estimated at the scale appropriate for a particular forensic analysis, and also different alternative contributors have different genetic backgrounds.

The origins of our study subjects are recorded at a national level, without reference to subnational ethnic identities. For example, in the analyses below Nigeria is treated as a subpopulation of a broader Afro-Caribbean population, but this ignores the substantial genetic variation among different groups within Nigeria. In forensic applications, it is appropriate to consider a distribution of 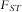 values over alternative possibilities for X. Because an LR involves in effect a product over loci with an 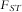 value applied at each locus, a single 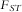 value for use in computing the LR should come from the upper tail of the 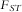 distribution. Below, we will report posterior median estimates of 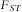, but when discussing forensic applications we will use the posterior 97.5 percentile, thus tending to over-estimate which is favourable to defendants.

We report 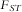 values that are much lower than have been obtained from single nucleotide polymorphisms (SNPs). This in part reflects the within-nation population mixing described above, but low 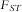 estimates also suggest a homogenising effect of STR mutation, which has previously been reported (Xu et al., 2000; Lu et al., 2012). It may also reflect that STRs employed in forensics were chosen in part on the basis of limited variation across populations, although many of the loci were chosen when little population data were available.

An extensive survey of worldwide human STR loci (Pemberton et al., 2013) focussed on well-defined ethnic groups, often with small population sizes, rather than the large and often ethnically mixed populations that are expected to be well represented in our database. Another recent study (Silva et al., 2012) has used worldwide forensic STR databases. We go beyond these papers in giving 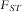 estimates at both within-continent and between-continent scales, and in using both observed and inferred reference populations. Our estimates are likelihood based, thus correctly account for variable sample size and provide posterior quantiles. They are directly relevant for forensic casework, and are also of broader interest in understanding human genetic variation in general populations at national, regional and continental scales.

## Materials and Methods

### Database

**Figure 1:**
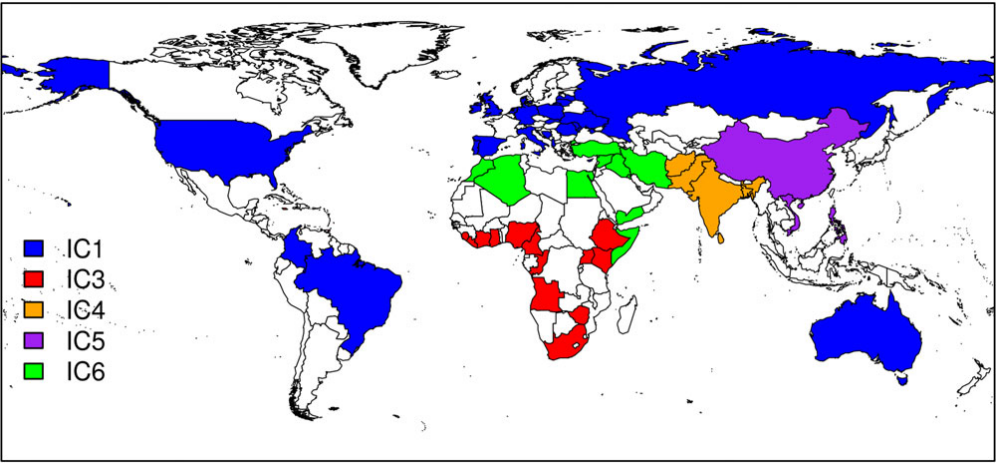
Countries of origin of the individuals included in the study, coloured according to the population that provides the best fit according to the indirect method (see text). White indicates countries represented by fewer than five individuals.

Our data set includes the STR profiles of 7 121 individuals living in the UK or Eire, or applying to migrate to the UK on the basis of relatedness to a UK resident. They are all genotyped by the same laboratory at up to 16 STR loci. The individuals are self identified into one of six populations: White (IC1 and IC2, with IC2 including darker-skinned individuals of European origin), Black African/Caribbean (IC3), South Asian (IC4), East/South-East Asian (IC5), or Middle Eastern/North African (IC6). They are further classified into subpopulations, in most cases defined at the national level. Our worldwide coverage is extensive (Fig. 1), but some large populations are not included, such as Japan and Indonesia, and the sample sizes from Latin America are small. Our analyses use only allele counts and not individual genotypes. In a few instances of only one allele observed at a locus, the peak intensity was insufficient to confirm homozygote status, leading to only one allele being recorded at that locus. Thus, total allele counts are not always even integers (Table 1).

**Table 1:** Number of alleles typed per locus and population. IC1-6 correspond to populations; Caucasian (IC1), Black African/Caribbean (IC3), South Asian (IC4), East/South-East Asian (IC5), and Middle Eastern/North African (IC6)

Observations	IC1	IC2	IC3	IC4	IC5	IC6	Total
D3S1358	7013	162	5200	704	625	226	13930
TH01	6953	158	5177	694	624	226	13832
D21S11	7006	162	5198	704	624	225	13919
D18S51	6944	157	5180	704	626	226	13837
D16S539	6951	162	5183	694	626	226	13842
VWA	7013	162	5194	704	626	226	13925
D8S1179	7007	162	5200	704	626	226	13925
FGA	6988	162	5196	700	626	226	13898
D19S433	6836	158	5122	687	621	226	13650
D2S1338	6575	152	4995	667	620	220	13229
D22S1045	1822	56	3478	523	506	162	6547
D1S1656	1835	56	3509	528	511	162	6601
D10S1248	1823	56	3497	516	506	118	6516
D2S441	1808	56	3458	521	501	160	6504
D12S391	1869	56	3531	551	507	162	6676
SE33	376	4	1039	308	396	140	2263

Subpopulations with >40 individuals sampled were included in our analyses. Some subpopulations of particular interest were also included despite having sample size <40. We merged or removed other subpopulations with small sample sizes. Study participants self identified both population and subpopulation labels, and in some cases we changed the population classification to better fit the subpopulation, as described below. These decisions require some subjective judgement; there is no canonical classification scheme for human populations.

#### IC1 and IC2

IC2 individuals from Europe were moved to IC1. Two national subpopulations were kept distinct, Eire and Great Britain, while the remaining European subpopulations were merged according to the United Nations geo-scheme for Europe (United Nations Statistics Division, 2014):

Eastern Europe: Hungary, Moldova, Poland, Romania, Russia, Slovakia, Ukraine.
Northern Europe: Denmark, Latvia, Lithuania, Sweden.
Southern Europe: Albania, Bosnia, Croatia, Cyprus, Greece, Italy, Kosovo, Malta, Macedonia, Portugal, Spain, Yugoslavia.
Western Europe: Belgium, France, Germany, Netherlands.

IC2 individuals from Argentina, Bolivia, Brazil, Columbia, Mexico, and Venezuela were combined (“Latin America”), as were IC1 individuals from Australia, New Zealand, and USA (“Anglo New World”). Those with no subpopulation identified, and those from Jersey, Northern Ireland, or South Africa, were removed.

#### IC3

Six national subpopulations were kept distinct: Ghana, Jamaica, Kenya, Nigeria, Sierra Leone, and Uganda. The following subpopulations were created from mergers according to the United Nations geo-scheme for Africa (United Nations Statistics Division, 2014), with Middle and Southern Africa combined as Central/Southern Africa:

Other W Africa: Benin, Gambia, Guinea, Guinea-Bissau, Ivory Coast, Liberia, Mali, Togo.

Other C/S Africa: Angola, Chad, Congo, Cameroon, South Africa.

Other E Africa: Burundi, Ethiopia, Eritrea, Malawi, Rwanda, Sudan, Tanzania, Zambia, Zimbabwe.

Other Caribbean: Barbados, Bermuda, Dominica, Guyana, Grenada, Monserrat, St Lucia, Virgin Islands, Trinidad.

Individuals with missing subpopulation were included as “Unknown IC3.” Those with origin not in Africa or the Caribbean were removed (Eire, GB, USA). Algeria, Egypt, Morocco, and Somalia were all included with IC6 (see “Best population fit” below).

#### IC4

Four national subpopulations were kept distinct: Afghanistan, Bangladesh, India, Pakistan. Individuals with missing subpopulation, or if the subpopulation was Nepal or Sri Lanka, were included as “Unknown IC4.” Mauritius was removed.

#### IC5

SE Asian subpopulations were merged (Cambodia, Indonesia, Philippines, Thailand, Vietnam). Mongolia and South Korea were merged with the much larger China sample to form NE Asia. Fiji was removed.

#### IC6

Iran, Iraq, Somalia, and Turkey were kept as separate national subpopulations. Other subpopulations were merged into N Africa (Algeria, Egypt, Morocco) or Middle East (Jordan, Kuwait, Lebanon, Palestine, Qatar, Syria, Yemen, UAE). Those from Georgia or with no subpopulation identified were removed. Afghanistan was moved to IC4.

The UK Forensic Science Service (FSS) previously collated (Foreman & Evett, 2001) databases of STR frequencies at 10 loci, in six populations with similar definitions to our data set: EA1 (Caucasian), EA2 (Mediterranean), EA3 (Afro-Caribbean), EA4 (South Asian), EA5 (East Asian), and EA6 (Middle East/North Africa). These databases are small (<2000 individuals combined) and do not include subpopulation labels. EA5 and EA6 both have sample sizes varying over loci, and the average sample size is reported below. Until recently, these were the reference databases used in most DNA forensics in the UK. Please note that the IC population codes refer to our new 16-locus data set, while the EA codes refer to the historic 10-locus data set.

#### Filtering Out Possible Relatives

Pairwise allele sharing was measured in all subpopulations, counting only loci for which both individuals were genotyped and including all pairs of individuals that had at least four genotyped loci in common. If >75% of alleles were shared, the individual with the fewest loci typed was removed. For subpopulations with <100 individuals, the threshold for removal was reduced to 50% allele sharing.

### Definition and Estimation of 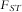


There are various ways to define, estimate and interpret 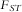 (Bhatia et al., 2013). The original definition (Wright, 1949) compared the variance of an allele fraction over subpopulations (S) to its variance in the total population (T):


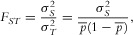
(1)

where 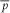 denotes the population allele fraction. The total population used in this formulation is usually a hypothetical ancestral population, from which observed subpopulations are assumed to have descended (Weir, 2001). However, in forensic work it is necessary to compare the subpopulation of a suspect with the population from which the available allele frequency database has been drawn. Thus, the reference population allele fractions are observed rather than inferred (Balding & Nichols, 1997). We will refer to these two approaches to estimation of 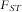 as the indirect and direct methods, respectively.

Moment-based estimators of 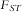 are widely used (Bhatia et al., 2013), but we take advantage of the benefits of likelihood-based estimation, which include high precision, correct accounting for sample size and interpretable intervals and quantiles (Balding, 2003, 2005). Weir & Hill (2002) proposed maximum likelihood estimation of 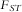 using a normal approximation to the multinomial, but the multinomial-Dirichlet (Mosimann, 1962) provides a natural likelihood without a large-sample assumption. Given a locus with *k* distinct alleles, the multinomial-Dirichlet has 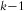 parameters specifying the population allele fractions, which are replaced with observed values in the direct method and are unknown parameters in the indirect method. The remaining parameter λ specifies the variance, and 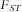 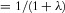. Throughout 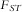 will be reported in percent.

#### Direct Method

The multinomial-Dirichlet likelihood is used for allele counts in a subpopulation, with reference allele fractions obtained from reference database counts, adjusted by adding a pseudocount of one for each allele in order to avoid zero values. The FSS databases EA1-6 are used as reference databases throughout. The direct analyses below only use the 10 loci in common between our data set and the historic FSS database, which are the loci with total allele counts >10^4^ (Table 1).

The likelihood curve for 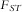 can automatically be interpreted as a posterior density with respect to a uniform prior. To formulate an informative prior, we noted previous work with small sample sizes (Balding & Nichols, 1997) suggesting that 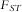 typically lies below 4%. Since more diverse subpopulations are considered here, we chose a beta prior distribution for 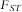, with median 2.3% and 95% credible interval (CI) from 0.26% to 8.0%.

**Figure 2:**
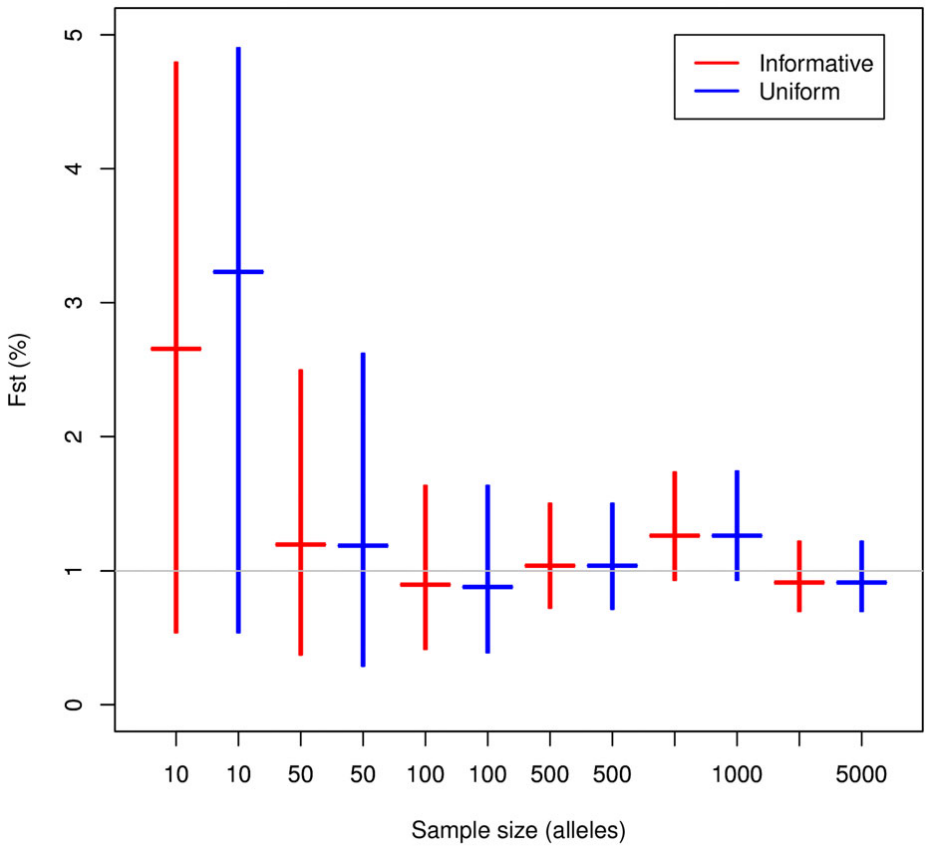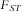 posterior 95% interval using: (red) a beta prior with median 2.3% and 95% CI (0.26%, 8.0%); (blue) the uniform prior. Sample sizes are shown on *x*-axis. Data were simulated to have 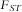 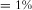 (horizontal line). The vertical lines indicate the 95% equal-tailed CI, and medians are indicated with horizontal segments.

To illustrate the effects of sample size, we performed direct estimation under both the uniform and beta priors using different sample sizes. Multinomial allele counts were simulated based on allele fractions that were Dirichlet-distributed, with means given by the EA4 allele fractions and 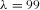 so that 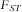 = 1%. The 95% CI includes 1% at all sample sizes, and becomes tighter as the sample size is increased ( Fig. 2). For small sample sizes, the beta prior leads to slightly smaller posterior interval widths than the uniform, and the posterior median moves towards the prior value.

**Figure 3:**
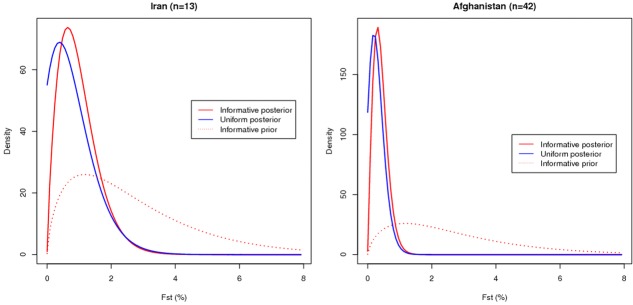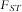 posterior densities (solid lines) using the direct method, given a uniform prior (blue) and an informative beta prior (red). Dotted red lines show the beta prior density. The subpopulations analysed are (left) Iran and (right) Afghanistan, with the reference populations being EA6 (Middle East/North Africa) and EA4 (South Asia), respectively.

Figure 3 shows that the choice of prior has a noticeable effect on the posterior for Iran (*n* = 13), and less so for Afghanistan (*n* = 42), in both cases the informative prior shifts the 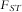 posterior distribution to slightly higher values compared with the uniform prior.

#### Indirect Method and Locus Dependence

The direct method is the most appropriate for forensic applications because the role of the reference database in 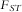 estimation matches its role in computing DNA profile likelihoods. The indirect method requires no reference database, so the 10-locus FSS databases are not used in these analyses and we are thus able to utilise 15 of the 16 available loci (SE33 is excluded due to low sample sizes, Table 1).

In the indirect method, the reference population is not observed, but is assumed to be a hypothetical ancestral population from which two or more observed subpopulations have descended independently. We used the BayesFST software (Beaumont & Balding, 2004) which implements a Markov Chain Monte Carlo method to sample from the posterior distribution of 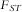 in each subpopulation given the allele counts. BayesFST assigns a jointly uniform prior distribution to the ancestral allele fractions at each locus, and uses the model


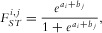
(2)

where 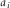 and 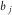 denote locus and population effects, respectively. All inferences reported below are based on 150 000 posterior values.

We first investigated the variation of 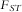 estimates across loci, treating IC1 through IC6 as six subpopulations of the hypothetical ancestral population. Each subpopulation parameter 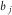 was assigned an N(−3, 1.8) prior, while the locus parameters 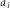 were assigned an N(0,1) prior. The resulting prior distribution for 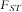 has a prior median 4.7%, with 95% CI from 0.02% to 92%. Table 2 shows that the posterior 95% CI for the 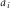 include zero for 13 of the 15 loci. In view of this limited evidence for locus heterogeneity, we subsequently set the locus effect parameter to be close to zero in order to estimate an average 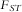 over loci and hence allow greater comparability across analyses. The implied prior median is then 4.7%, with 95% CI from 0.1% to 63%.

**Table 2:** Posterior 95% intervals for locus effect parameters using the indirect method. The analysis used all 7121 individuals with IC1 through IC6 treated as six subpopulations

	Percentile			Percentile	
			
Locus	2.5	97.5	Locus	2.5	97.5
D3	−1.72	−0.2	D19	−0.62	0.62
TH01	0.11	1.58	D2	−0.59	0.62
D21	−0.85	0.45	D22	−0.06	1.32
D18	−0.79	0.38	D1	−0.7	0.52
D16	−1.3	0.15	D10	−0.87	0.6
vWA	−0.93	0.42	D2	−0.21	1.15
D8	−0.73	0.6	D12	−0.71	0.56
FGA	−1.04	0.23			

We repeated all analyses with only the 10 loci used in the direct analyses, and confirmed that resulting inferences were similar, but on average more precise with 15 loci (10-locus results not shown). Thus, the differences reported below between direct and indirect 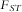 values for a subpopulation are almost entirely due to the different reference population, rather than the different number of loci used.

#### Best Population Fit

Each subpopulation defined above was assigned to the FSS database giving the “best fit” (lowest median 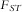 under the direct method), for both direct and indirect method analyses below. The majority of allocations were as expected: most European subpopulations fit best with EA1, most African and Caribbean subpopulations with EA3, all South Asian subpopulations fit best with EA4, both East Asian subpopulations fit best with EA5 and most Arab subpopulations fit best with EA6. Three subpopulations close to the Middle East fit EA6 equally or slightly better than their nominal population: Southern Europe (EA1), Afghanistan (EA4) and Kenya (EA3). The nominal classification was retained in each case.

One discrepancy was much larger: Somalia fit better with EA6 (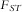
=1.5%) than with the nominal EA3 (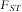
=2.2%), and we subsequently included Somalia with IC6. Although Somalia borders Kenya (EA3), it is also geographically close to the Arab world, and there have historically been many links. Mitochondrial (Mikkelsen et al., 2012) and Y-chromosome (Sanchez et al., 2005) studies have both suggested a strong Arab influence in Somali genetics, although their highest similarity is usually with neighbouring Eastern Ethiopians and Northern Kenyans. HLA typing (Mohamoud, 2006) also suggests that Somalis are more similar to Arabs than to sub-Saharan Africans. Pickrell et al. (2014) estimate the Eurasian ancestry of Somalis at roughly 38% using admixture mapping, supporting the low 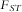 estimate for Somalia with the EA6 database.

## RESULTS

### EA1

When comparing subpopulations to the EA1 reference population (Table 3), all the European subpopulations have an 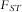 estimate (97.5 percentile) under 1%, except Western Europe, which has the smallest sample size. The low 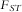 estimate for Southern Europe supports the merging of European-origin IC2 individuals with IC1, suggesting that IC2 might usefully be redefined to only include Latin Americans with predominantly European ancestry. The Anglo New World has slightly lower estimates than Western Europe, but Latin America has a higher 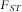 estimate, presumably due to admixture with non-European populations.

**Table 3:** The 2.5, 50, and 97.5 posterior percentiles of 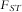 (expressed as %). Subpopulations were compared both individually with the reference population EA1 (direct method, 10 loci) and analysed jointly to infer ancestral allele fractions (indirect method, 15 loci). *n* denotes the sample size (number of individuals)

		Direct			Indirect		
			
IC1	*n*	2.5	50	97.5	2.5	50	97.5
Eire	1949	0.1	0.2	0.2	0.0	0.0	0.1
Great Britain	1416	0.1	0.1	0.1	0.0	0.0	0.0
Eastern Europe	61	0.2	0.5	1.0	0.1	0.3	0.7
Northern Europe	45	0.0	0.3	0.8	0.0	0.2	0.5
Southern Europe	60	0.0	0.2	0.5	0.0	0.1	0.3
Western Europe	13	0.1	0.7	2.1	0.0	0.5	1.8
Anglo New World	13	0.1	0.5	1.7	0.0	0.3	1.4
Latin America	25	0.5	1.3	2.4	0.6	1.3	2.4

The indirect method gives lower 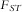 estimates than the direct method, which is expected because the ancestral allele fractions are inferred to be towards the centre of the subpopulation values. However, the 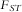 values for Latin America are almost unchanged and are again the highest, because inference of ancestral allele fractions is dominated by the European populations.

### EA3

The mixed subpopulations of West, Central-Southern and East Africa, as well as Unknown IC3, have lower 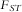 estimates under the direct method than the national subpopulations of Ghana, Kenya, Nigeria, and Sierra Leone. The 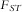 estimate for other Caribbean is high, much higher than for Jamaica. Jamaicans have a predominantly African origin (Caribbean Community Capacity Development Programme, 2009), and there are approximately 800 000 people of Jamaican descent living in the UK (International Organisation for Migration, 2007), which is close to half the UK population categorised as black (Office for National Statistics, 2011). Therefore the EA3 database may be expected to include a large number of Jamaicans.

Indirect estimation (Table 4b) gives noticeably different results than the direct method. In most cases they are greatly reduced, the exception being Kenya which is geographically remote from the majority of subpopulations, which are in West Africa or the Caribbean. We have noted above that Kenya fits almost equally well with both EA3 and EA6 using direct estimation, suggesting some genetic influence from the Arab world.

**Table 4:** The 2.5, 50, and 97.5 posterior percentiles of 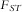 (expressed as %). Subpopulations were compared both individually with the reference population EA3 (direct method, 10 loci) and analysed jointly to infer ancestral allele fractions (indirect method, 15 loci). *n* denotes the sample size (number of individuals)

		Direct			Indirect		
			
IC3	*n*	2.5	50	97.5	2.5	50	97.5
Ghana	214	0.8	1.1	1.6	0.2	0.3	0.5
Jamaica	166	0.5	0.7	1.0	0.0	0.1	0.2
Kenya	51	0.7	1.2	1.9	0.8	1.3	1.9
Nigeria	444	0.9	1.2	1.5	0.2	0.3	0.3
Sierra Leone	41	0.7	1.3	2.2	0.1	0.3	0.8
Uganda	63	0.3	0.5	1.0	0.0	0.2	0.4
Unknown IC3	864	0.4	0.5	0.7	0.0	0.0	0.0
Other Caribbean	20	0.5	1.5	2.9	0.1	0.4	1.3
Other C/S Africa	55	0.3	0.6	1.1	0.0	0.1	0.3
Other E Africa	66	0.3	0.7	1.1	0.0	0.1	0.4
Other W Africa	48	0.1	0.5	1.0	0.0	0.1	0.3

### EA4, EA5, and EA6

For EA4 and EA5, the 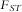 estimates are all low for both direct and indirect methods, with no outliers (Tables 5 and 6). The 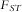 estimates for India and Bangladesh are much lower for the indirect than the direct method. The 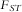 estimate for NE Asia is higher than that for SE Asia using the direct method, but lower using the direct method. This suggests the EA5 database largely consists of individuals from NE Asia.

**Table 5:** The 2.5, 50, and 97.5 posterior percentiles of 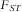 (expressed as %). Subpopulations were compared both individually with the reference population EA4 (direct method, 10 loci) and analysed jointly to infer ancestral allele fractions (indirect method, 15 loci). *n* denotes the sample size (number of individuals)

		Direct			Indirect		
			
IC4	*n*	2.5	50	97.5	2.5	50	97.5
Afghanistan	47	0.1	0.3	0.9	0.1	0.4	0.9
Bangladesh	53	0.1	0.4	0.9	0.0	0.1	0.4
India	49	0.0	0.3	0.8	0.0	0.1	0.4
Pakistan	60	0.0	0.2	0.5	0.0	0.2	0.5
Unknown IC4	76	0.0	0.2	0.5	0.0	0.1	0.2

**Table 6:** The 2.5, 50, and 97.5 posterior percentiles of 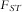 (expressed as %). Subpopulations were compared both individually with the reference population EA5 (direct method, 10 loci) and analysed jointly to infer ancestral allele fractions (indirect method, 15 loci). *n* denotes the sample size (number of individuals)

		Direct			Indirect		
			
IC5	*n*	2.5	50	97.5	2.5	50	97.5
NE Asia	260	0.1	0.2	0.3	0.1	0.4	0.8
SE Asia	44	0.0	0.2	0.7	0.0	0.1	0.4

Most IC6 subpopulations have low sample sizes, and so we will here discuss the posterior median of 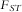 rather than the 97.5 percentile. Iraq has low 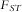 estimates, much lower than its neighbour Iran (Table 7). Unsurprisingly, large 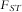 estimates were obtained for Somalia. Results are largely congruent between the direct and indirect method, however, Turkey has a larger 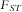 estimate using the indirect method, which may be due to Turkish individuals being well represented in the EA6 database.

**Table 7:** The 2.5, 50, and 97.5 posterior percentiles of 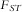 (expressed as %). Subpopulations were compared both individually with the reference population EA6 (direct method, 10 loci) and analysed jointly to infer ancestral allele fractions (indirect method, 15 loci). *n* denotes the sample size (number of individuals)

		Direct			Indirect		
			
IC6	*n*	2.5	50	97.5	2.5	50	97.5
Iran	12	0.1	0.9	2.4	0.1	0.9	2.7
Iraq	28	0.0	0.2	0.7	0.0	0.2	0.7
Somalia	494	1.1	1.3	1.7	1.2	1.6	2.1
Turkey	20	0.1	0.5	1.6	0.2	0.9	2.1
Middle East	24	0.1	0.7	1.8	0.1	0.5	1.6
N Africa	26	0.2	0.7	1.7	0.1	0.6	1.5

### Fringe Regions

We use the term “fringe” for subpopulations that have similar affinity to two populations (difference in median 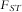 <0.001). Broadly speaking these regions reflect an overall smooth change in allele frequencies with geography, so that the fringe regions are at the boundaries of our continental-scale populations (Table 8). Thus, Afghanistan is near the boundary between IC4 and IC6, and fits them approximately equally well, S Europe is at the boundary between IC1 and IC6, and Kenya is the IC3 country nearest to IC6. These results suggest a relatively low differentiation between IC6 and all three surrounding populations (IC1, IC3, IC4). Only IC5 is not linked to other populations through a fringe subpopulation, perhaps due to the mountains separating China from South Asia, and its geographical remoteness from IC1 and IC3. This agrees with a previous report that East Asian populations are distinct from those of South Asia, but are close to South East Asian populations (HUGO Pan-Asian SNP Consortium, 2009).

**Table 8:** Posterior median 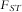 (%) for fringe subpopulations: These are subpopulations for which another reference population gives a median 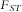 estimate using the direct method within 0.001 of the lowest (best fit) value

	Reference				
	
Fringe	EA1	EA3	EA4	EA5	EA6
Afghanistan	1.17	2.90	0.78	1.87	0.78
Kenya	2.32	1.39	2.51	2.32	1.36
Southern Europe	0.30	2.99	1.20	2.03	0.34
Unknown IC4	1.68	2.80	0.62	1.17	0.72

### Inter-Population Comparisons

Above we have compared subpopulations with continental-scale reference populations, and now we make comparisons among those populations. Each column of Table 9 shows a different 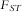 analysis of the five IC populations, using an EA database as the reference database in the direct method, or using the indirect method.

**Table 9:** Posterior median 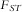 (%):Populations IC1-6 were compared to each reference population in turn using the direct method. The indirect method was used to compare each population to a hypothetical global ancestral population

		Reference					
		
Global	*n*	EA1	EA3	EA4	EA5	EA6	Indirect
IC1	3582	0.4	3.1	1.9	1.9	0.9	2.7
IC3	2032	1.7	0.7	1.7	1.4	1.1	1.0
IC4	285	1.4	3.1	0.7	1.3	0.8	2.3
IC5	304	3.1	4.2	2.4	0.5	2.0	3.3
IC6	604	1.8	1.7	1.9	1.7	0.9	1.4

For the direct method, each IC database showed the best fit (lowest 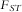 estimate) with its cognate EA database, reflecting a reasonable consistency of definitions between IC and EA databases. The highest 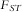 value for IC1, IC4 and IC5 are all obtained relative to EA3. Conversely, looking down the columns of Table 9, IC5 shows the highest 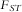 value for each EA database except EA5. The IC6 database is influenced by the large sample size from Somalia, and shows similar 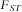 values with respect to all four EA databases other than EA6.

Using indirect estimation, IC3 and IC6 show the lowest 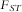 values, while IC5 shows the highest value, corresponding to an inferred ancestral human population similar to that of modern North-East Africa (Pemberton et al., 2013).

## Discussion

Although we have only examined 10 or 15 STR loci in this study, their multi-allelic nature and the large sample sizes for many subpopulations means that we have been able to achieve good precision in many of the 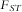 estimates that we report, as indicated by the 95% posterior intervals. We have shown that 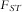 estimates depend sensitively on the choice of reference population, and in particular that the use of a population reference database can generate very different 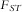 estimates from those based on a hypothetical ancestral population, which is the usual practice in population genetic studies.

Silva et al. (2012) collated STR databases worldwide, and reported a global 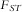 estimate from forensic data sets of 2.3%, comparable with inter-population estimates reported here (Table 9), while the corresponding estimate from the nonforensic Human Genome Diversity Project (HGDP) data set was more than twice as high, at 5.3%. Silva et al. suggest that this discrepancy is due to forensic markers being selected to have low differentiation among populations. However, they also demonstrate that selecting high heterozygosity markers decreases 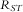, and current forensic markers were selected in part to achieve high heterozygosity. The difference may also reflect larger and more ethnically mixed populations being included in forensic surveys, while the HGDP data set includes many ethnically distinct populations, often of small size.

Nelis et al. (2009) used the HapMap SNP database (before the upgrade to HapMap 3) to estimate continental genetic distance between Africa, Asia, and Europe. The 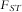 values ranged from 11% (Europeans compared with Asians) to 19% (Africans compared with Asians), much higher than the STR-based estimates reported here and in Silva et al. (2012). This may be due to the high STR mutation rate (Weber & Wong, 1993) tending to stabilise allele fractions across populations, for example through mutations in short alleles tending to favour expansion, while contractions are favoured in long alleles (Sibly et al., 2003; Dupuy et al., 2004; Lu et al., 2012). Excoffier & Hamilton (2003) demonstrated that the discrepancy between 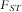 estimates from SNP markers and those from STR markers can be removed by taking into account the stepwise mutation seen at STR markers. However, the broad pattern of variation is similar for STRs as for SNPs (Ramachandran et al., 2005; Pemberton et al., 2013).

One motivation for this research is to guide forensic practice, and overall we find that 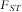 ⩽ 3% should be appropriate for most forensic calculations. The 97.5 posterior percentile for 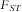 lies under 3% for all subpopulations relative to their best fit population, consistent with more limited previous results (Balding & Nichols, 1997; Gill et al., 2003). Low values can be justified in some settings, for example 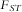 = 1% appears adequate for Asians (both South and East), but 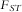 = 3% would be more robust against incorrect assignment of reference population for an unknown contributor. In some cases it may be possible to tailor the 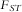 value to specific circumstances, for example a lower 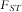 value may be appropriate for alternative contributors who are known to be Jamaican, rather than from another Caribbean island.
